# A Novel Organo-Selenium Bandage that Inhibits Biofilm Development in a Wound by Gram-Positive and Gram-Negative Wound Pathogens

**DOI:** 10.3390/antibiotics3030435

**Published:** 2014-08-25

**Authors:** Phat L. Tran, Saurabh Patel, Abdul N. Hamood, Tyler Enos, Thomas Mosley, Courtney Jarvis, Akash Desai, Pamela Lin, Ted W. Reid

**Affiliations:** 1 Departments of Ophthalmology and Visual Sciences, School of Medicine, Texas Tech University Health Sciences Center, Lubbock, TX 79430, USA; E-Mails: phat.tran@ttuhsc.edu (P.L.T.); thomas.mosley@yahoo.com (T.M.); courtney.jarvis@ttuhsc.edu (C.J.); akash.desai@ttuhsc.edu (A.D.); pamela.lin@ttuhsc.edu (P.L.); 2 Department of Neonatology, University of Illinois, Chicago, IL 60607, USA; E-Mail: drsaurabh.14@gmail.com; 3 Departments of Medical Microbiology and Immunology, Texas Tech University Health Sciences Center, Lubbock, TX 79430, USA; E-Mail: abdul.hamood@ttuhsc.edu; 4 South Western Medical Center, University of Texas, Dallas, TX 75390, USA; E-Mail: tyler.enos@utsouthwestern.edu

**Keywords:** biofilm formation, wound healing, organo-selenium, bandage

## Abstract

Biofilm formation in wounds is a serious problem which inhibits proper wound healing. One possible contributor to biofilm formation in a wound is the bacteria growing within the overlying bandage. To test this mechanism, we used bandages that contained a coating of organo-selenium that was covalently attached to the bandage. We tested the ability of this coating to kill bacteria on the bandage and in the underlying tissue. The bandage material was tested with both lab strains and clinical isolates of *Staphylococcus aureu*s, *Pseudomonas aeruginosa* and *Staphylococcus epidermidis.* It was found that the organo-selenium coated bandage showed inhibition, of biofilm formation on the bandage *in vitro* (7–8 logs), with all the different bacteria tested, at selenium concentrations in the coating of less than 1.0%. These coatings were found to remain stable for over one month in aqueous solution, 15 min in boiling water, and over 6 years at room temperature. The bandages were also tested on a mouse wound model where the bacteria were injected between the bandage and the wound. Not only did the selenium bandage inhibit biofilm formation in the bandage, but it also inhibited biofilm formation in the wound tissue. Since selenium does not leave the bandage, this would appear to support the idea that a major player in wound biofilm formation is bacteria which grows in the overlying bandage.

## 1. Introduction

Bacterial infections in wounds are difficult to treat and can be found in patients with diabetes [1], surgical wounds [2,3], traumatic wounds [4] and burns [5]. The two most common bacteria that cause wound infections are *Pseudomonas aeruginosa* and *Staphylococcus* *aureus* [1,2,3,4,5]. These are opportunistic pathogens, which do not cause many problems in healthy individuals; however, they can readily cause infections in individuals in which the host defenses are affected. A good example of this is seen in burn patients when the tissue is damaged [5]. These bacteria can enter the wound from the normal flora of the patient or a health care worker, as seen in the cases of *S. aureus*, and can be acquired from the environment, as seen in the cases of *P. aeruginosa*. These organisms can thrive in the environment of a wound. In burn wounds, both *P. aeruginosa* and *S. aureus* often exist as biofilms [6,7].

A biofilm is a community of bacterial cells which can be formed from different bacteria organized to perform different functions [8]. For instance, *P. aeruginosa* displays multiple phenotypes during development as a biofilm [9]. Also, *P. aeruginosa* biofilms form in a set of different phases [9]. The first phase is a reversible attachment which is followed by the second phase of irreversible attachment of bacteria to strata, which can be formed from fragments of tissue, a matrix, cellular and/or artificial. This is followed by different stages of maturation where the bacterial cells form protective layers around themselves (these layers can be composed of compounds such as lipopolysaccharide—LPS). Ultimately, the biofilm enters a dispersive phase where bacteria can be shed into the surrounding tissue [9]. This ability of the biofilm to form a barrier towards antibodies and blood cells presents a serious medical management problem. In addition, since many of the bacterial cells in the biofilm are in a quiescent state, they are very resistant to antibiotics [8]. This is why it is important to have a covering over a wound that provides a barrier to bacteria, and at the same time, it does not provide the bacteria with a platform for bacterial biofilm formation.

Currently, many bandages contain silver which is embedded in the substrate of the material, and these silver bandages have been shown to prevent bacterial infection [10,11,12,13]. However, silver compounds (the most common compound at the present time) must leach from the bandage, and they are toxic to human fibroblasts at even low concentrations [14,15]. In addition, these compounds have limited half-lives because of their leaching requirement. If the silver compounds are in a high enough concentration to be effective, they also tend to blacken the wound from the formation of elemental silver, which makes it difficult for the physician to gauge the healing progression of the wound. In addition, tests of different silver bandages only show around four logs of killing [16]; however, organo-selenium as described below, offers an alternative.

It has been shown that organo-selenium can be covalently attached to many types of surfaces and still generate superoxide radicals (O_2_^•^¯) as it has been shown in the past with different types of materials [17,18,19,20,21]. The di-selenide compounds, used in the synthesis of the coating, are reduced by thiols (sulfhydryl groups, R-SH) which can be found in physiological fluids, and form selenide anions, R-Se^−^ [22]. This can be seen in Equation (1) below. The catalytic selenide anion, R-Se^−^, then reacts with oxygen to form O_2_^•^¯ and a putative thiyl radical R-Se^•^. R-Se^• ^then reacts with two moles of thiol (supplied in the assay and *in vivo* as glutathione [GSH]) to regenerate the original selenide anion as shown in the Equations (2) and (3) below [22]. Equations (2) and (3) form a catalytic mechanism since the original selenide anion is regenerated.

R-Se-Se-R + 2 GSH → 2R-Se^−^ + GSSG (1)

R-Se^−^+ 2O_2_→ R-Se^•^+ 2O_2_^•^¯ (2)

R-Se• + 2 GSH → R-Se^−^ + GSSG (3)

While this catalytic ability of selenium has been known since 1947 [23], the pro-oxidative characteristics of selenium compounds were not elucidated until 1989 by Seko *et al.* [24]. Since the mechanism used by selenium is catalytic and the antimicrobial effect is caused by the generated superoxide radical, the organo-selenium compound does not have to leave the surface of the bandage. This means there is no tissue toxicity.

Recently, we showed that organo-selenium compounds could be covalently bound to solid matrixes, and they retained their ability to catalyze the formation of superoxide radicals to inhibit bacterial attachment to a surface [17,18,19,20,21]. The present study looks at the ability of an organo-selenium bandage to not only block the ability of both *P. aeruginosa* and *S. aureus* to form a biofilm on the bandage material, but also to inhibit biofilm formation in the underlying wound.

## 2. Results and Discussion

### 2.1. Organo-Selenium Bandage, Coated with Different Organo-Selenium Concentrations, was Tested for Its Ability to Block Biofilm Formation in Vitro for Laboratory Strains of Staphylococcus aureus and Pseudomonas aeruginosa

**Figure 1:**
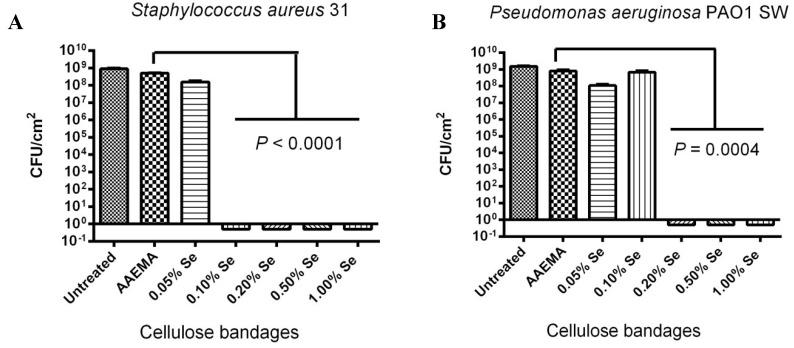
Dose response of an organo-selenium coated bandage on the inhibition of bacterial biofilm formation. The colony forming unit (CFU) determination is for a 1 cm^2^ bandage in 1 mL of solution. (**A**) *S. aureus* 31; (**B**) *P. aeruginosa* PAO1 SW.

Using the biofilm inhibition assay (CFU assay), the organo-selenium bandage was tested against lab strains of *S. aureu*s and *P. aeruginosa*, added to bandages with different selenium concentrations. The bandages were coated with organo-selenium in the form of Se-AAEMA. As can be seen in Figure 1A, over eight logs of inhibition were seen for *S. aureus* 31 biofilm formation at 0.1% selenium concentration. In addition, 0.05% selenium showed only a little over a log of inhibition when it is compared with the bandage coated with the control AAEMA containing no selenium.

For *P. aeruginosa* PAO1 SW, (Figure 1B), the bandage materials, coated with different concentrations of selenium, showed over eight logs of inhibition (100%) at 0.2% selenium. In addition, 0.1% selenium showed less logs of inhibition when it was compared with control bandage coated with AAEMA containing no selenium.

These results show that Gram-negative bacteria are slightly more resistant to inhibition of biofilm formation by the selenium-catalyzed formation of superoxide. However, both Gram-negative and Gram-positive bacteria were inhibited (>8 logs of inhibition) at low (<0.2%) organo-selenium concentrations.

### 2.2. Confocal Visualization of the Biofilm Inhibition on a Bandage by Organo-Selenium in Vitro

**Figure 2:**
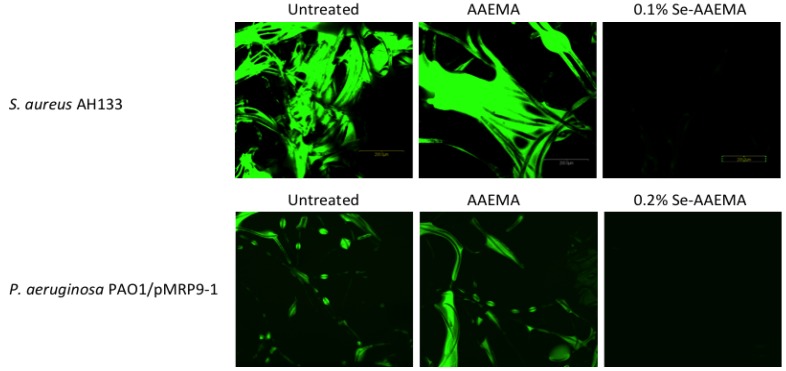
CLSM picture of biofilm development by *S. aureus* AH133 and *P. aeruginosa* PAO1/pMRP9-1 on a bandage with different treatments.

In order to visualize the biofilm formation, confocal images of the biofilm at different selenium concentrations were carried out using the Confocal Laser Scanning Microscope (CSLM). Bandages from the same study, as was carried out above, were used. As seen in Figure 2, it shows a dramatic difference between the Se-AAEMA coated bandage (which has no biofilm—lack of fluorescence) and the control bandage with no treatment or AAEMA bandage with the AAEMA alone (which have biofilms as seen from the fluorescence). The fluorescence is due to the GFP produced in the bacteria.

The above CLSM pictures of different biofilms show that the organo-selenium treatments were quite effective in the inhibition of biofilm formation for both *S. aureus* and *P. aeruginosa*, and they are consistent with the results of the CFU determinations in Figure 1*.*

### 2.3. Selenium Bandage Inhibition of Clinical Isolates of S. aureus, P. aeruginosa and S. epidermidis

**Figure 3:**
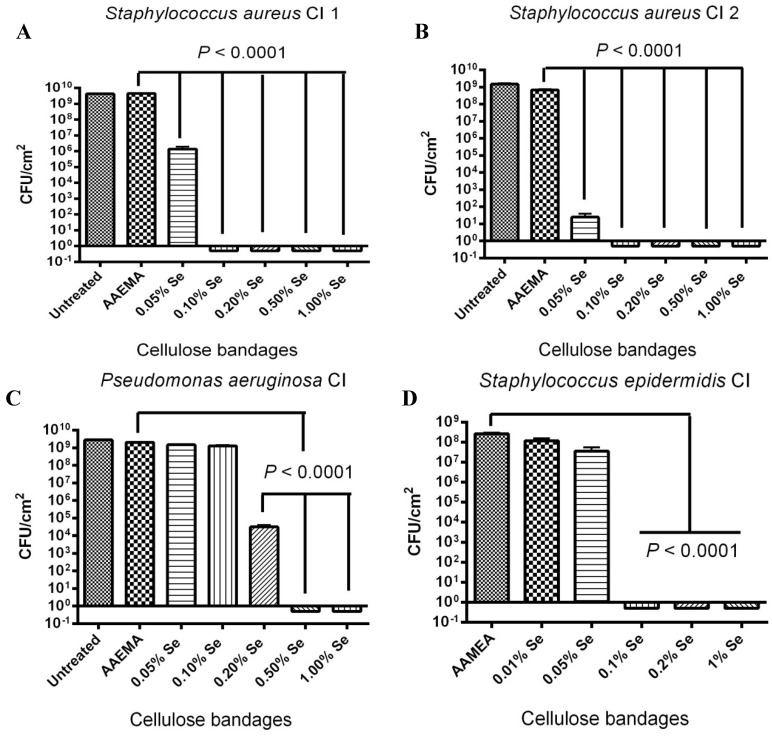
Dose response of an organo-selenium coated bandage on the inhibition of biofilm formation by different clinical isolate strains. The CFU determination is for a 1 cm^2^ bandage in 1 mL of solution. (**A**) *S. aureus* Cl 1; (**B**) *S. aureus* Cl 2; (**C**) *P. aeruginosa* Cl; (**D**) *S. epidermidis* Cl.

Additional studies were carried out to see if the organo-selenium coated bandages were also able to inhibit clinical strains of the different bacteria as well as it did for the laboratory strains. As shown in Figure 3A,B, the inhibition of the *S. aureus* clinical isolates 1 and 2 was quite effective. Approximately, nine logs of inhibition were observed for 0.1% selenium in both strains; however, the two strains did show some differences at lower selenium concentrations. Strain 1 showed only four logs of inhibition with 0.05% Se-AAEMA while strain 2 showed over seven logs of inhibition with 0.05% Se-AAEMA. Interestingly, both strains showed better inhibition at 0.05% Se-AAEMA than the laboratory strain (Figure 1).

A similar study was carried out with clinical isolates of *P. aeruginosa* and *S. epidermidis*. *P. aeruginosa* clinical isolate showed that the selenium treated bandages were not as effective in biofilm inhibition as compared to the laboratory strain of *P. aeruginosa*., nine logs of inhibition were not obtained until 0.5% Se-AAEMA (Figure 3C), while approximately nine logs was obtained at 0.2% with the laboratory strain (Figure 1B).

A study was carried out with a clinical isolate of *S. epidermidis*, and the results are seen in Figure 3D. Similar to those results seen for *S. aureus*, there were eight logs of inhibition of biofilm formation are seen at 0.1% Se-AAEMA.

The above results of over eight logs of inhibition can be compared with the ability of silver bandages to inhibit biofilm formation. A study of several different types of silver bandages showed that the best bandages only exhibited 4–5 logs of inhibition of biofilm formation with different bacterial strains [16].

### 2.4. Stability of the Selenium Bandage in Preventing Biofilm Formation

**Figure 4:**
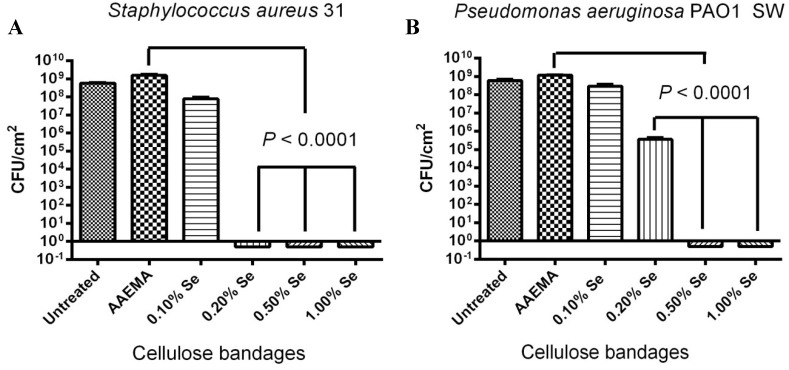
One month stability study of the selenium bandage bacterial biofilm formation. The CFU determination is for a 1 cm^2^ bandage in 1 mL of solution. (**A**) *S. aureus* 31; (**B**) *P. aeruginosa* PAO1 SW*.*

A stability study was carried out to see if the organo-selenium coated bandages retained their biofilm inhibitory activity after one month of storage in PBS solution at 37 °C. As seen in Figure 4, there was some loss of inhibitory activity; however, there was still eight logs inhibition against *S. aureus* 31 at 0.2% Se-AAEMA (Figure 4A) and 8 logs inhibition against *P. aeruginosa*, at 0.5% Se-AAEMA (Figure 4B).

The overlying solution from bandages of the one-month stability studies was analyzed for the selenium and no free selenium was detected in the solution. The detection limit was 0.001 ug/mL.This is consistent with the stability data which still showed eight logs of inhibition of biofilm formation after soaking in PBS for one month.

**Figure 5:**
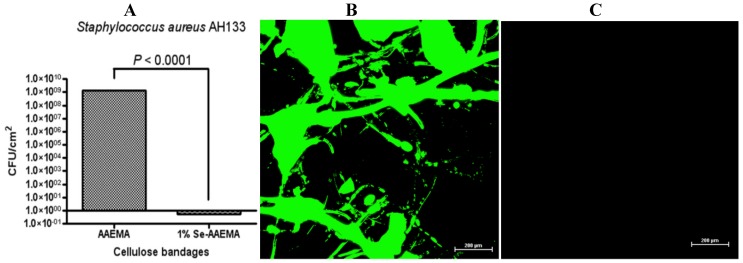
Six-year stability study of the selenium bandage bacterial biofilm formation. The CFU determination is for a 1 cm^2^ bandage in 1 mL of solution. (**A**) Ability of an organo-selenium (1%) coated bandage (which set on a shelf at room temperature for over 6 years) to inhibit *S. aureus* AH1333 biofilm formation. CLSM study of the *in vitro* inhibition of a *S. aureus* AH133 bacterial biofilm in a bandage coated with (**B**) AAEMA and with (**C**) Se-AAEMA after setting on a shelf at room temperature for over 6 years.

Bandages material coated with Se-AAEMA (1%) was also allowed to set dry at room temperature for 6 years, and they were then tested with the bacterial CFU assay and the confocal laser scanning microscopy. These results can be seen below in Figure 5.

From the CFU analysis, over eight logs of biofilm inhibition was obtained. Also, the CLSM analysis confirmed the results. Thus, the shelf-life at room temperature for this material appears to be longer than 6 years.

### 2.5. Stability of the Organo-Selenium Bandage to Boiling Water

**Figure 6:**
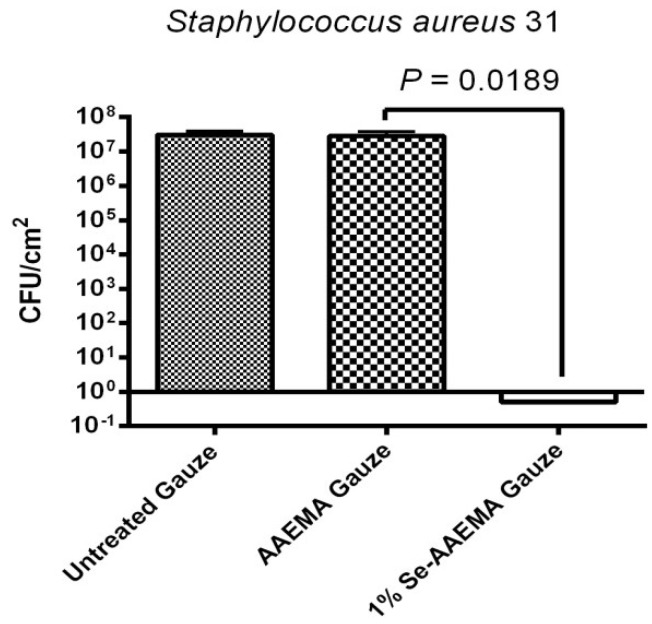
Stability of *S. aureus* 31 biofilm inhibition by the selenium bandage after boiling in water for 15 min. The CFU assay is for a 1 cm^2^ bandage in 1 mL of solution.

A study was carried out to see if the organo-selenium bandage would still be stable after submersion in boiling water for 15 min. The boiling temperature was performed because it is important to test the stability of the bandages at higher temperatures due to higher environmental temperatures experienced in the developing world. As seen in Figure 6, over seven logs of inhibition were seen against *S. aureus* 31 at 1% selenium after the boiling water treatment. 

### 2.6. In Vivo Study of the Ability of a 1% Selenium Coated Bandage to Inhibit Biofilm in Both the Bandage and the Underlying Wound

**Figure 7:**
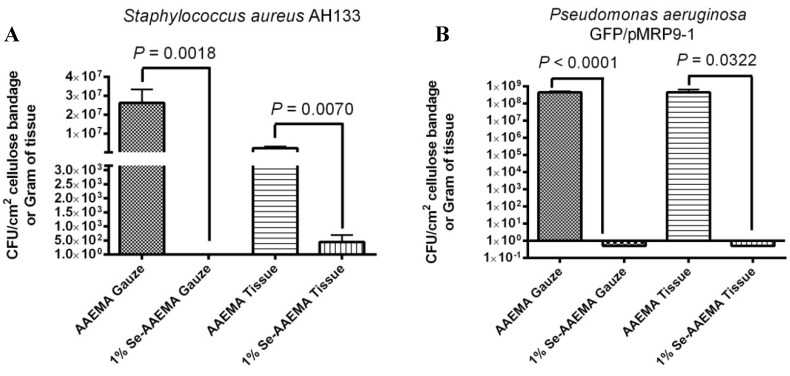
*In vivo* inhibition of a *S. aureus* AH1333 bacterial biofilm in both the bandage and underlying tissue. The CFU assay is for a 1 cm^2^ bandage or per gram of tissue.

*In vivo* studies were carried out using a mouse wound model. A 1 cm^2^ wound on the back of a mouse was injected with 10^3^–10^4^ bacteria under the bandage, and the experiments were allowed to set for 3 days. At the end of that time, the bandage was removed, and the tissue under the bandage was dissected. Both the bandage and tissue from the wound were then homogenized, and a bacterial colony forming unit assay was carried out. Figure 7 showed the quantitative results for the 1% Se-AAEMA coated bandage with *S. aureus* AH133 (A) and *P. aeruginosa* GFP/pMRP9-1 (B). The control bandage was coated with AAEMA with no selenium. As seen from Figure 7A, over seven logs of inhibition was seen in the organo-selenium bandage, and six logs of inhibition with almost complete inhibition was seen in the underlying tissue when compared with the controls. A similar *in vivo* study was carried out with *P. aeruginosa* GFP. As seen in Figure 7B, inhibition (over eight logs) was obtained in both the 1% selenium bandage and the underlying tissue. The 1% Se-AAEMA bandage was used, since preliminary results showed that 0.2% and 0.5% were not as effective *in vivo*.

**Figure 8:**
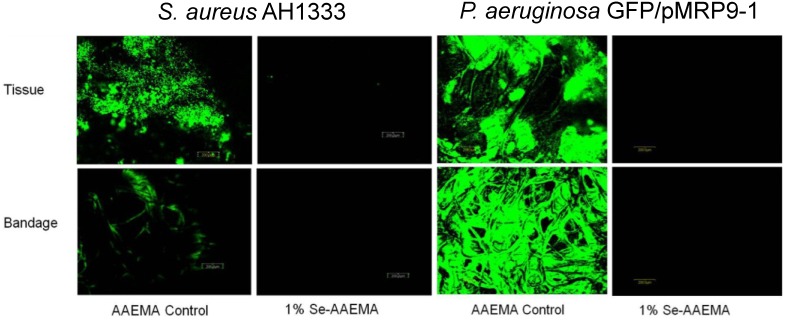
CLSM study of the *in vivo* inhibition of a *S. aureus* AH1333 (**A**) and *P. aeruginosa* GFP/pMRP9-1 (**B**) bacterial biofilm in both the bandage and underlying tissue.

Visualization of the CFU results from Figure 7 with the confocal laser scanning microscope (CLSM) confirming these results, as seen in Figure 8. 

The *in vivo* results for the *S. aureus* AH1333 and *P. aeruginosa* GFP/pMRP9-1 studies were also examined by CLSM (Figure 8), and they were found to agree with the results above in Figure 7. In both the tissue and the bandage, there were over eight logs of inhibition by the 1% selenium coated bandage.

This *in vivo* data showed that the organo-selenium coating on the bandage causes 7–8 logs of inhibition of biofilm formation in the bandage, and it also causes essentially the same amount of inhibition of biofilm formation in the wound. This would tend to indicate that it is necessary for the bandage to serve as a reservoir for the bacteria, so it can more easily infect the wound. A wound on a normal mouse has many defense mechanisms to kill bacteria that enter the wound. However, a biofilm that has developed in a bandage over the wound, offers the potential for the bacteria to overwhelm these normal defenses and establish an infection in the wound. This is consistent with the data since the selenium is covalently attached to the bandage and cannot leave the bandage. The fact that the bandage is in contact with the wound should still allow the bacteria to form a biofilm in the wound. Because the half-life of the superoxide radical is very short (microseconds), the ability of the radicals to travel into the wound is taken away. This is why no tissue damage was seen in the wounds, and it was not observed on the cornea cells underlying a contact lens coated with selenium after two months in a rabbit eye [16]. Thus, no biofilm was seen under the Se-AAEMA bandage, but the biofilm was observed under the control bandage.

### 2.7. Mammalian Cell Toxicity Assay

To check for the possibility that selenium was leaching from the bandage, Se-AAEMA coated cellulose was soaked in PBS for a month and the toxicity of the solution to a mammalian monolayer L929 of mouse fibroblast cells, using the Agar diffusion test (performed by Toxikon Corp, Bedford, MA, USA), was examined. Positive control solutions showed severe reactivity (Grade 4) at a 48 h observation while the negative control and the Se-AAEMA disc extract solutions showed no signs of reactivity (Grade 0) by the cell cultures exposed for 48 h. Thus, the solution around the bandage was not toxic to mammalian cells after soaking the Se-AAEMA bandage for one month. This is consistent with the results obtained in Section 2.4 where no selenium (less than 0.01 μg/mL) was detected by trace analysis in the one month leaching solution.

## 3. Experimental

### 3.1. Coating Cellulose Gauze with Polymerized Se-AAEMA

The Se-AAEMA monomer was purchased from Eburon Organics International (catalog No. 700.010; Lubbock, TX, USA). Se-AAEMA (22% [wt/wt] selenium) stock solution was dissolved and diluted in 99.99% 2-(methacryloyloxy)ethyl acetoacetate (AAEMA) to yield different concentrations of the Se-AAEMA (wt/wt). Hospital grade rolled gauze bandage (LOT1783A, Johnson and Johnson, New Brunswick, NJ, USA) were cut in 4 inches × 4 inches square pieces and soaked in sterile distilled water overnight. The washed gauze bandages were then dried and coated with AAEMA or Se-AAEMA. Briefly, the bandages were soaked in AAEMA or Se-AAEMA for 30 min. Excess AAEMA or Se-AAEMA was removed from the bandages by passing the soaked bandage through a roller press. The AAEMA or Se-AAEMA bandages were then sprayed with H_2_O_2_ using a bottle sprayer to initiate free radical polymerization. This has little or no effect on the bandage material. As a result of the *in situ* polymerization, the polymer is held in place by a combination of van der Waals forces and physical interlinking with the bandage material. The bandages were then transferred to a 66 °C curing oven. After curing, the bandages were washed twice (1 h each) in sterile phosphate-buffered saline (PBS, pH 7.4) at 37 °C. The bandages were then dried, cut into 1 and 1.5 square centimeter pieces, sterilized by dried autoclaving, dried again, and stored at room temperature until used in the assays. All percentages listed for the different concentrations are percent selenium and do not represent the percentage of the total organo-selenium compound which would be approximately twice as high.

### 3.2. Bacterial Strains

All strains were grown in Luria Bertani (LB) Broth, Mueller Hinton Broth, or on LB Agar plates at 37 °C. The *Staphylococcus aureus* strain ATCC 31 was obtained from Remel (Lenexa, KS, USA). The prototrophic *Pseudomonas aeruginosa* strain PAO1 SW, originally isolated from an infected wound [25], was obtained from S. E. H. West (University of Wisconsin, Madison). *S. aureus* AH133, a lab strain that constitutively expresses green fluorescent protein from a plasmid (pCM11) in the presence of 1 μg/mL erythromycin [26]. *Pseudomonas aeruginosa* strain PAO1/pMRP9-1. This strain carries the plasmid pMRP9-1 that contains the gene that codes for the green fluorescent protein [17]. To maintain the plasmid, the strain was grown in the presence of 300 μg/mL carbenicillin. The *S. aureus*, *P. aeruginosa*, and *S. epidermidis* clinical isolates were obtained from leg or abscess wounds from the Clinical lab at Texas Tech University Health Sciences Center under an approved Institutional Review Board protocol, Texas Tech University Medical center/Lubbock, Texas. All bacterial stock was stored at −80 °C.

### 3.3. Colony-Forming Unit Assays—in Vitro

The biofilm assay was performed as previously described with some modifications [17]. Bacteria were grown overnight, washed once with PBS (pH 7.4), re-suspended in PBS (pH 7.4) to an optical density (OD_600_) of 0.5 (10^8^ CFU/mL), and serially diluted 10-fold. All PBS assay solutions contained 150 μM reduced glutathione (Sigma Chemical, St. Louis, MO, USA). One hundred micro liter aliquots containing 10^2^–10^3^ colony-forming units were added to either a one square centimeter AAEMA (control) bandage, or Se-AAEMA test bandage. After the bacterial inoculum was absorbed into the bandages, they were placed on LB agar plates, and the plates were incubated at 37 °C for 24 h. Biofilms were quantified by determining the CFU per square centimeter of bandage*.* Following incubation, each bandage piece was gently washed twice with sterile PBS to remove any planktonic bacteria. Excess PBS was drained from the bandage by touching it to a sterile filter paper and the bandage was then transferred to a sterile 1.5-mL micro centrifuge tube containing 1 mL of PBS for enumeration of bacteria. The tubes were placed in a water bath sonicator for 10 min to loosen the cells within the biofilm and then vigorously vortexed 3 times for 1 min to detach the cells. Suspended cells were serially diluted (10-fold) in PBS, and 10-μL aliquots of each dilution were spotted onto LB Agar plates. In experiments where no bacteria was detected, a 100 μL zero dilution sample was tested. Thus, the equation for back calculating the bacterial concentration was CFU × dilution factor × 100, with the exception of the 100 μL sample which was calculated as CFU × dilution factor × 10. This means that the smallest amount of bacteria that we could detect would be approximately 10 bacteria. All experiments were done in triplicate, and all measurements were repeated at least three times. We used this data to calculate the CFU injected under the bandages for the *in vivo* experiments.

### 3.4. Mouse Wound Infection Model

Six adult female Balb C mice (including a control group) weighing 20–24 g were anesthetized using a mixture of isoflurane and oxygen and their backs were shaved. Shaved areas were completely cleansed with 95% ethanol. A flap of skin was lifted and a 1.0 cm^2^ piece of skin was removed centrally in the shaved area with sterile scissors. Either AAEMA control or Se-AAEMA bandages (1.5 cm^2^) was placed on the wound. The bandages were secured in place by applying a clear OPSITE dressing over the back of the mouse. Aliquots containing 10^3^–10^4^ CFU of bacteria in 50 μL were injected in the area between the bandage and the wound. The mice were monitored twice a day for signs of infection or distress. After 72 h of observation, the mice were euthanized. The bandages were removed, and the connective tissue around the wound and the tissue under the wound (down to the spinal cord) was then dissected and removed. The extracted bandages and all the dissected tissue were gently rinsed in PBS and homogenized in PBS. Excess PBS was drained from the bandage by touching it to a sterile filter paper and the bandage was then transferred to a sterile homogenized tube containing 2 mL of PBS for enumeration of bacteria by either determination of CFU or by confocal microscopy. To determine CFU counts, the bandages were homogenized to loosen the cells within the biofilm and then vigorously vortexed 3 times for 1 min to detach the bacterial cells. Suspended cells were serially diluted (10-fold) in PBS, and 10-μL aliquots of each dilution were spotted onto LB Agar plates. All experiments were done at least in triplicate.

Tissue: At the end of the experiment (72 h), the mouse was euthanatized and the connective tissue around the wound and the tissue under the wound (down to the spinal cord) was then dissected and removed. This was then homogenized and used for a CFU assay in a manner similar to that used for the bandage above.

Animals were treated in accordance with the protocol approved by the Institutional Animal Care and Use Committee at Texas Tech University Health Sciences Center in Lubbock, TX, USA. 

### 3.5. Biofilm Analysis by Fluorescence Microscopy

The pieces of bandage at the end of the 72 h incubation were assayed as described above for the CFU assays. Six AAEMA control and six Se-AAEMA bandage segments were also examined for the presence of biofilm with Confocal Laser Scanning Microscopy (CLSM) using an Olympus Fluoview FV300 (Olympus America, Center Valley, PA, USA). Animals were treated in accordance with the protocol approved by the Institutional Animal Care and Use Committee at Texas Tech University Health Sciences Center in Lubbock, TX, USA.

### 3.6. Analysis of the Long-term Stability of the Organo-Selenium-Containing Gauze Bandage

One square-centimeter of the organo-selenium-containing gauze bandages were completely immersed in 1.0 mL PBS in sterile glass tubes and incubated at 37 °C for a month. At the end of the incubation period, the pieces were removed, dried, sterilized by dried autoclaving, and utilized in the *in vitro* biofilm assay, as described above, for determination of the durability of the anti-biofilm activity of the coating. The overlying solution was analyzed for free selenium by an independent laboratory (Trace Analysis Inc, Lubbock, TX, USA). This was carried out to show that the selenium bonds were stable to hydrolysis and that the selenium did not leave the bandage. In addition, a bandage sample coated with Se-AAEMA that set dry at room temperature for 6 years was also tested by the bacterial CFU assay.

### 3.7. Accessing the Stability of the Organo-Selenium-Containing Gauze Bandage at Elevated Temperature

One square-centimeter of the organo-selenium-containing gauze bandages was completely immersed in 10.0 mL PBS in sterile glass tubes and boiled for 15 min. At the end of the boiling period, the pieces were removed, dried, sterilized by dried autoclaving, and utilized in the *in vitro* biofilm assay, as described above, for determination of the stability of the coating at boiling temperature.

### 3.8. Analysis of Selenium Toxicity to Mammalian Cells

The assay was conducted using a commercially available service (Toxikon Corporation, Bedford, MA, USA). Briefly, the biological reactivity of a mammalian monolayer, L929 mouse fibroblast cell culture, in response to the one month soaking solutions obtained from 0.2% selenium coated discs (Se-AAEMA) was determined. The mammalian monolayer was protected from mechanical damage, while allowing diffusion of leachable chemicals from the one month soaking solutions, with an overlay of agar stained with a vital dye (neutral red). One hundred μL of soaking solutions was placed in a sterile filter disc with a surface area ≥100 mm^2^. The soaking solution was applied directly to the surface of the agar, in duplicate. Positive (Buma-N-Rubber) and negative (Negative Control Plastic) controls were prepared to verify the proper functioning of the test system. The cultures were incubated at 37 ± 1 °C, in a humidified atmosphere containing 5% ± 1% carbon dioxide, for 48 h. Biological reactivity (cellular degeneration and malfunction) was rated on a scale from Grade 0 (No reactivity) to Grade 4 (Severe reactivity). The soaking solutions met the requirement of the test (not considered toxic) if none of the cultures exposed to the soaking solutions showed greater than a Mild Reactivity, Grade 2.

### 3.9. Statistical Analysis

Results of the CFU assays were analyzed with Prism^®^ version 4.03 (GraphPad Software, San Diego, CA, USA) with 95% confidence intervals (CIs) of the difference. Comparisons of the *in vivo* biofilms formed on Se-free and Se-AAEMA bandages were analyzed by a two-tailed unpaired *t*-test to determine significant differences. All experiments were done at least in triplicate.

## 4. Conclusions

It was found that the organo-selenium (Se-AAEMA) coated bandage showed inhibition of *Staphylococcus aureu*s and *Pseudomonas aeruginosa* biofilms *in vitro* at less than 0.2% selenium with laboratory strains. *Staphylococcus aureu*s and *Staphylococcus epidermidis* clinical strains showed inhibition of biofilm formation at less than 0.1% selenium while *Pseudomonas aeruginosa* clinical strain showed inhibition at less than 0.5% selenium*.*

In stability studies, it was shown that the organo-selenium coated bandage retained its ability to inhibit biofilm formation after soaking for a month at 37 °C, in PBS, for 15 min in boiling water and for over 6 months at room temperature dry on the shelf. Thus, the organo-selenium was stable in the bandage.

*In vivo* studies were carried out using a mouse wound model where *Staphylococcus aureu*s or *Pseudomonas aeruginosa* was injected between the organo-selenium bandage and the wound and allowed to grow for three days. It was found that not only did the organo-selenium coating block biofilm formation in the bandage but it also blocked it in the wound. In this study, it was shown that the organo-selenium molecules did not come off the bandage, (they are covalently attached). Also, since the selenium atoms catalyze the formation of superoxide radicals on the surface of the bandage, and superoxide has a very short half-life, both the selenium and the superoxide radicals would not be able to migrate into the wound. The fact that biofilm was found under the control bandage but not under the selenium bandage would imply that the bandage acts as a reservoir for free bacteria to form a wound biofilm, and is an important mechanism for development of a wound infection. Bacterial biofilm formed in the bandage would appear to allow the bacteria to overwhelm the normal defense mechanisms in the wound.

## References

[1] Lipsky B.A., Berendt A.R., Deery H.G., Embil J.M., Joseph W.S., Karchmer A.W., Lefrock J.L., Lew D.P., Mader J.T., Norden C. (2004). Diagnosis and treatment of diabetic foot infections. Clin. Infect. Dis..

[2] Markogiannakis H., Pachylaki N., Samara E., Kalderi M., Minettou M., Toutouza M., Toutouzas K.G., Theodorou D., Katsaragakis S. (2009). Infections in a surgical intensive care unit of a university hospital in Greece. Int. J. Infect. Dis..

[3] McCaig L.F., McDonald L.C., Mandal S., Jernigan D.B. (2006). *Staphylococcus aureus*-associated skin and soft tissue infections in ambulatory care. Emerg. Infect. Dis..

[4] Murray C.K. (2008). Infectious disease complications of combat-related injuries. Crit. Care Med..

[5] Church D., Elsayed S., Reid O., Winston B., Lindsay R. (2006). Burn wound infections. Clin. Microbiol. Rev..

[6] Schaber J.A., Triffo W.J., Suh S.J., Oliver J.W., Hastert M.C., Griswold J.A., Auer M., Hamood A.N., Rumbaugh K.P. (2007). *Pseudomonas aeruginosa* forms biofilms in acute infection independent of cell-to-cell signaling. Infect. Immun..

[7] Trafny E.A. (1998). Susceptibility of adherent organisms from Pseudomonas aeruginosa and *Staphylococcus aureus* strains isolated from burn wounds to antimicrobial agents. Int. J. Antimicrob. Agents.

[8] Donlan R.M., Costerton J.W. (2002). Biofilms: Survival mechanisms of clinically relevant microorganisms. Clin. Microbiol. Rev..

[9] Sauer K., Camper A.K., Ehrlich G.D., Costerton J.W., Davies D.G. (2002). *Pseudomonas aeruginosa* displays multiple phenotypes during development as a biofilm. J. Bacteriol..

[10] Holder I.A., Durkee P., Supp A.P., Boyce S.T. (2003). Assessment of a silver-coated barrier dressing for potential use with skin grafts on excised burns. Burns.

[11] Kuroyanagi Y., Shiraishi A., Shirasaki Y., Nakakita N., Yasutomi Y., Takano Y., Shioya N. (1994). Development of a new wound dressing with antimicrobial delivery capability. Wound Repair Regen..

[12] Lin S.S., Ueng S.W., Lee S.S., Chan E.C., Chen K.T., Yang C.Y., Chen C.Y., Chan Y.S. (1999). *In vitro* elution of antibiotic from antibiotic-impregnated biodegradable calcium alginate wound dressing. J. Trauma.

[13] Ong S.Y., Wu J., Moochhala S.M., Tan M.-H., Lu J. (2008). Development of a chitosan-based wound dressing with improved hemostatic and antimicrobial properties. Biomaterials.

[14] Hidalgo E., Bartolomé R., Barroso C., Moreno A., Domínguez C. (1998). Silver nitrate: Antimicrobial activity related to cytotoxicity in cultured human fibroblasts. Skin Pharmacol. Appl. Skin Physiol..

[15] Lee A.R., Moon H.K. (2003). Effect of topically applied silver sulfadiazine on fibroblast cell proliferation and biomechanical properties of the wound. Arch. Pharm. Res..

[16] Ip M., Lui S.L., Poon V.K.M., Lung I., Burd A. (2006). Antimicrobial activities of silver dressings: An *in vitro* comparison. J. Med. Microbiol..

[17] Mathews S.M., Spallholz J.E., Grimson M.J., Dubielzig R.R., Gray T., Reid T.W. (2006). Prevention of bacterial colonization of contact lenses with covalently attached selenium and effects on the rabbit cornea. Cornea.

[18] Tran P.L., Hammond A.A., Mosley T., Cortez J., Gray T., Colmer-Hamood J.A., Shashtri M., Spallholz J.E., Hamood A.N., Reid T.W. (2009). Organoselenium coating on cellulose inhibits the formation of biofilms by *Pseudomonas aeruginosa* and *Staphylococcus aureus*. Appl. Environ. Microbiol..

[19] Tran P.L., Lowry N., Campbell T., Reid T.W., Webster D.R., Tobin E., Aslani A., Mosley T., Dertien J., Colmer-Hamood J.A. (2012). An organoselenium compound inhibits *Staphylococcus*
*aureus* biofilms on hemodialysis catheters *in vivo*. Antimicrob. Agents Chemother..

[20] Tran P., Hamood A., Mosley T., Gray T., Jarvis C., Webster D., Amaechi B., Enos T., Reid T. (2013). Organo-selenium-containing dental sealant inhibits bacterial biofilm. J. Dent. Res..

[21] Wang J.C., Tran P.L., Hanes R., Cordero J., Marchbanks J., Reid T.W., Colmer-Hamood J.A., Hamood A.N. (2013). Inhibition of otopathogenic biofilms by organoselenium-coated tympanostomy tubes. JAMA Otolaryngol. Head Neck Surg..

[22] Chaudiere J., Courtin O., Leclaire J. (1992). Glutathione oxidase activity of selenocystamine: A mechanistic study. Arch. Biochem. Biophys..

[23] Feigl F., West P.W. (1947). Test for selenium based on catalytic effect. Anal. Chem..

[24] Seko Y., Imura N. (1997). Active oxygen generation as a possible mechanism of selenium toxicity. Biomed. Environ. Sci..

[25] Holloway B.W., Krishnapillai V., Morgan A.F. (1979). Chromosomal genetics of* Pseudomonas*. Microbiol. Rev..

[26] Malone C.L., Boles B.R., Lauderdale K.J., Thoendel M., Kavanaugh J.S., Horswill A.R. (2009). Fluorescent reporters for *Staphylococcus aureus*. J. Microbiol. Methods.

